# The Mechanical Properties of Direct Metal Laser Sintered Thin-Walled Maraging Steel (MS1) Elements

**DOI:** 10.3390/ma16134699

**Published:** 2023-06-29

**Authors:** Jerzy Bochnia, Tomasz Kozior, Jarosław Zyz

**Affiliations:** Faculty of Mechatronics and Mechanical Engineering, Kielce University of Technology, 25-314 Kielce, Poland; jbochnia@tu.kielce.pl (J.B.); jaroslawzyz@gmail.com (J.Z.)

**Keywords:** thin-walled elements, direct metal laser sintering (DMLS), mechanica properties, maraging steel

## Abstract

The aim of this study was to explore the mechanical properties of thin-walled maraging steel (MS1) elements fabricated using direct metal laser sintering (DMLS). This article first explains the fabrication procedure and then analyzes the results of the static tensile strength tests and microscopic (SEM) examinations. From this study, it is evident that the mechanical properties of such objects, particularly their tensile strength, are not affected by the build direction; no significant anisotropy was found. The experiments confirm, however, that the mechanical properties of thin-walled elements fabricated from MS1 by DMLS are largely dependent on thickness. The microscopic images of such elements show local discontinuities in the macrostructure of the molten material (powder). Although the research described here mainly contributes to the field of additive manufacturing, it also considers some aspects of Lean manufacturing.

## 1. Introduction

Additive manufacturing (AM) has long been used by the manufacturing sector for rapid prototyping applications [1]; yet, these days, it is increasingly employed to fabricate finished goods. The most common AM processes used for industrial purposes are powder bed fusion (PBF) technologies. Obviously, being able to withstand higher loading, metal powders are preferred to polymer powders. The research and development sector also makes intensive use of additive manufacturing, especially to create parts with complex geometries, which would be difficult or impossible to achieve using traditional manufacturing processes. The advances in AM include the development of new materials, with properties that need to be determined in detail before they are used in industry.

Despite the presence of 3D printing in the industry for over 40 years, there is a very big problem related to the construction and production of thin-walled models, including those with a cellular structure. As the research conducted by the authors of earlier articles [2,3,4] showed, the mechanical properties of thin-walled models produced with the use of 3D-printing technology largely differ from those determined for solid models with determined quality [5]. This problem results in the lack of guidelines related to the design of thin-walled models with predicted strength. In this work, both materials based on pure PLA and those reinforced with carbon fiber and the addition of bronze were analyzed. The results of the research showed clear differences and allowed for the determination of new, increased safety factors in the design and manufacture of thin-walled models for practical industrial use. In connection with this article, based on the results of our own work, an attempt was made to determine the strength of thin-walled models produced by 3D printing, though using materials based on metal powders. The novelty of the conducted research is the use of 3D-printing technology, which is well-known, to produce models with a characteristic structure at the limit of what is technologically possible and with properties outside the range declared by the manufacturers. In addition, the results can be implemented to simulate, for example, thin-walled cellular structures, which is perfectly suitable for applied science.

Examples of AM processes include electron beam melting (EBM) and selective laser melting (SLM), both enabling unconventional design solutions. The use of EBM to create thin-walled lightweight structures, such as double-wishbone suspensions, was described in [6]. The studied material was high-density Ti-6Al-4V alloy, and the experiments involved determining its mechanical properties and analysing its microstructure. It was found that the surface texture of thin-walled elements with a thickness of less than 2 mm had an effect on their mechanical properties. The mechanical properties, particularly fatigue strength, of stainless steel 316 L additively manufactured by SLM were discussed in [7]. The microscopic examinations showed that the fatigue damage was due to laser spatter, incomplete fusion, or the presence of multiple pores and other flaws that contributed to the propagation of cracks. The fatigue strength was also analyzed in [8]; here, SLM was used for the refurbishment of worn parts.

Additive manufacturing technologies are constantly being improved to eliminate the structural defects of parts. This can be achieved, for instance, by enhancing control systems, as proposed in [9], where the authors discussed some simulation-based feed-forward control models for process optimization.

The geometric accuracy and surface texture of toothed wheels fabricated by DMLS from high-chromium stainless steel (9GP1) using an EOS M270 printer were examined in [10]. The surface texture analysis was based on the measurement of selected two- and three-dimensional surface roughness parameters.

Some studies concentrated on the mechanical behavior of materials additively manufactured from metal powders [11,12,13,14]. The research described in [12], for instance, involved comparing the mechanical properties of four metal alloys fabricated by SLM or DMLS with those of similar materials conventionally manufactured by forging or machining. The fatigue strength was reported to be lower for AlSi10Mg fabricated by SLM or Ti6Al4V and made by DMLS than for the equivalent materials undergoing traditional manufacturing processes. The lower fatigue strength of the additively manufactured elements was due to the occurrence of multiple fatigue cracks initiated at surface flaws, internal voids, and microcracks. It was suggested that the fatigue strength of such parts could be improved by using heat treatment or hot isostatic pressing. From other studies, for example, that described the mechanical properties of elements fabricated by Laser Powder Bed Fusion (LPBF) from 18Ni-300 maraging steel powder [13], it is also evident that heat treatment is recommended for additively manufactured parts.

The behavior of 18Ni-300 maraging steel elements fabricated by LPBF was also analyzed in [15]. The experimental data confirmed the influence of the build direction on the part’s microstructure, mechanical properties, and character of deformations. The specimens were built in the horizontal and vertical directions. Uniaxial tensile strength tests were performed to compare the mechanical properties of as-fabricated elements with those subjected to subsequent heat treatment. Horizontally oriented specimens were observed to have higher tensile strength and plasticity than those built vertically. Another important finding was that heat treatment reduced the effect of the build orientation. The investigations were carried out on thick-walled (solid-type) specimens with a diameter of 6 mm. The physical properties of maraging steels were examined in [13,14,15,16].

Much of the research in this area was devoted to the design, fabrication, and properties of thin-walled elements, as recently these have been in higher demand [6,17,18,19,20,21]. Some interesting findings were discussed in [6]; they concerned the relationship between the thickness and tensile strength of Ti-6Al-4Vl alloy specimens fabricated by EBM. These experiments aimed to determine the tensile strength, modulus of elasticity, and elongation of specimens differing in thickness (0.5, 1, 1.5, 2, 3, or 6 mm). All three parameters were reported to increase with increasing specimen thickness. For the 0.5 mm thick models, they were 458.4 MPa, 59.7 GPa, and 2%, respectively, while for the 6 mm thick parts, the respective values were 922.8 MPa, 110.8 MPa, and 8.8%, respectively. These results can be useful for designers of thin-walled structures. Other issues related to the design and fabrication of thin-walled structures, including mathematical simulations, were analyzed in [19].

A positive effect of heat treatment on the mechanical properties and microstructure of additively manufactured elements was also confirmed in [22,23]. It should be mentioned here that 3D-printed polymer parts may also be subjected to appropriate heat treatment [24], which has a direct influence on the quality of printed models.

This article deals with direct metal laser sintering (DMLS) used to fabricate thin-walled specimens from maraging steel (MS1) powder. The purpose of this research is to present the differences regarding the mechanical properties of thin-walled models in relation to the data declared by the material’s manufacturer. In addition, one of the objectives is also to compare the strength of thin-walled samples of different thicknesses manufactured with the same technological parameters. An additional goal is to analyze the influence of the printing direction set on two levels, which allows for referring to the test results in the context of anisotropy and the design process of, e.g., a cell structure. This article focuses on their mechanical properties, i.e., tensile strength and the percentage of elongation after fracture. The research described here primarily contributes to the field of additive manufacturing. The findings, however, may also be of practical use to engineers specializing in process optimization (Lean manufacturing) or surface finishing.

## 2. Materials and Methods

### 2.1. Method

The experiments consisted of

- fabricating specimens using DMLS;
- measuring the dimensions;
- performing static tensile strength tests;
- examining the specimen structure using scanning electron microscopy (SEM).

The additive manufacturing technology selected for the study, i.e., DMLS, is used to create models from metal powders. According to the ISO/ASTM 52900 standard [25], this method is classified as a *powder bed fusion* (PBF) process, in which thermal energy is required to selectively fuse powder material to build a solid part. DMLS uses focused energy, e.g., laser energy, to bind metallic particles, so subsequent layers fully adhere to the layers below. The scanning is done in a localized fashion in the build chamber of the 3D printer. The metal powder material used in DMLS is applied layer by layer on the build platform. Each layer, spread evenly with a leveling roller, is sintered following a predefined scan (contour and hatch/fill) pattern. After the molten material solidifies to form a homogeneous structure, the platform drops by one layer, another layer of powder is applied, and the process begins again. After the part is finished, it is removed from the build platform for post-processing. It has specific physical properties, especially mechanical properties, specific geometric dimensions, and specific surface texture. The excess, i.e., unsintered, powder is removed to be reused. Due to the very small thickness of the samples, which is typical for thin-walled models (1–2 mm), there are certain limitations regarding the technological process. It was necessary to create additional support for the samples in a vertical position, which is not the case with solid models. The method of manufacturing thin-walled models also has limitations related to the minimum thickness of the built layer and the method of controlling, for example, the distribution of the filament or the path of a laser beam. Previous research [4] showed errors in the software related to the filling of thin-walled models, which should also be analyzed when generating G-code before using 3D-printing technology.

### 2.2. Materials

The specimens were built in an EOS M290 printer from EOS MaragingSteel MS1 powder. The mechanical properties provided in the material data sheet [26] are shown in Table 1.

### 2.3. Fabrication of the Specimens

**Figure 1:**
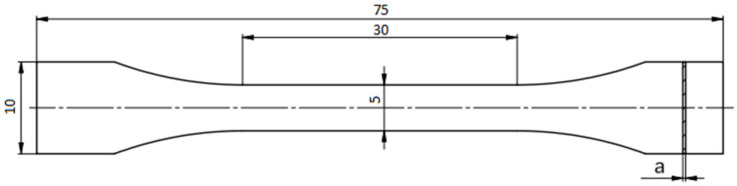
Geometric dimensions of the test pieces with a representing thickness (0.35, 0.40, 0.45, 0.50, 0.55, or 0.60 mm).

A 3D CAD program was used to create the solid models. Their dimensions are shown in Figure 1.

Once the specimen models were stored in *.stl* format, the files were verified in the Materialise *Magics* program; then, the specimens were properly arranged on the virtual platform, and, for the vertically oriented models, support structures were generated. Supports are generally required to avoid deformation of overhanging or protruding features under gravity and thermal load. Support material prevents a vertically built model from collapsing. It is also designed to reduce residual stress that occurs during the part’s cooling process. It is assumed that all downward facing surfaces or those inclined at an angle of less than 40° to the horizontal axis need to be supported with special structures. Before the files were exported to the virtual build platform of the printer, the verified models and the generated supports were saved in .*stl* format.

**Figure 2:**
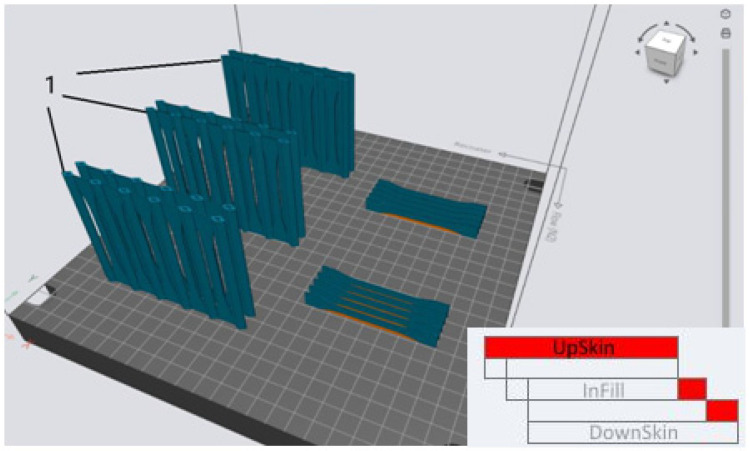
Example of a virtual arrangement of models on the build platform of the EOS M290 printer. 1—Specially designed supports to hold the vertically built thin-walled specimens in place.

The files prepared in the Materialise *Magics* program were exported to the EOSPRINT software, which made it possible to properly arrange the specimens and support material on the build platform. The files were then saved in .*openjz* format to be exported to the built-in computer of the EOS M290 printer. Finally, the files obtained for the specimens were linked with those for the special support structures, which were designed to hold the thin-walled specimens in place during fabrication. Figure 2 shows an example arrangement of specimens on the virtual platform.

The samples were made using the following technological printing parameters:

For Unskin: laser power 165 W, laser speed 675 mm/s, and hatch distance 0.09 mm resulting in energy density equal to 54.32 J/mm^3^;

For DownSkin: laser power 150 W, laser speed 2000 mm/s, and hatch distance 0.06 mm resulting in energy density equal to 25 J/mm^3^;

For Infill: laser power 305 W, laser speed 1010 mm/s, and hatch distance 0.11 mm resulting in energy density equal to 54.91 J/mm^3^.

**Figure 3:**
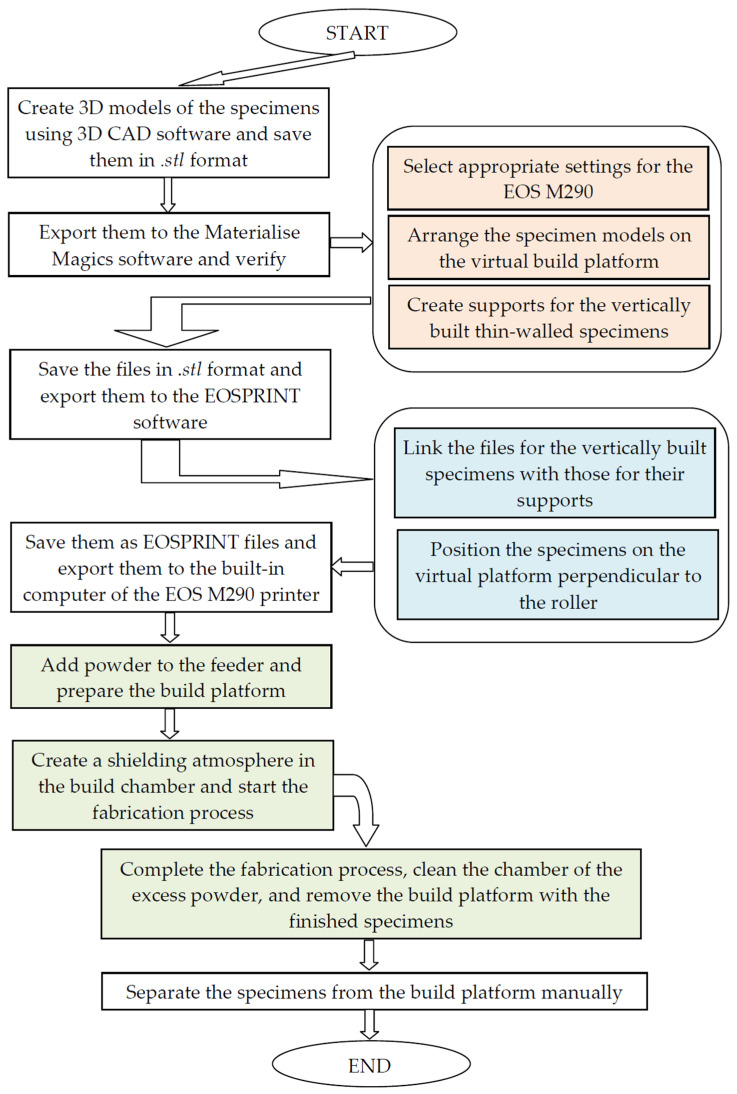
Specimen fabrication procedure.

The diagram representing the specimen fabrication procedure is shown in Figure 3.

**Figure 4:**
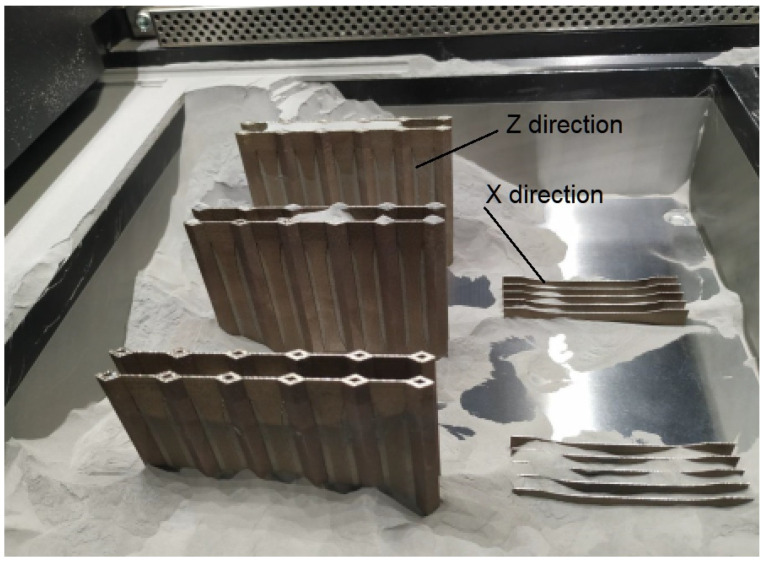
View of the build chamber with partly cleaned models.

Once the sintering was completed, and the build chamber was cooled, the parts were removed, separated, and cleaned. Unmolten powder went into the excess powder collector to be reused. Figure 4 shows the build chamber with unsintered powder partially removed. As the horizontally oriented specimens did not require much support material, they were removed and manually separated. The vertically built specimens, on the other hand, with a large amount of support material, had to be wire-cut from the platform using an electrical discharge machine.

After the specimens were separated from the platform and the supports, they were prepared for tensile tests. These required measuring their geometries and making some marks along the gauge length. Thin-walled specimens with thicknesses of 0.35, 0.40, or 0.45 mm could not be built in the vertical direction because vertical stability could not be achieved even with the use of special support structures.

### 2.4. Dimensional Measurement

Each specimen was measured with an accuracy of 0.01 mm at three points along the gauge length to determine the average thickness, $\bar{a}$, and the average width, $\bar{b}$. The measurement results are provided in Table 2. The average thickness and width data obtained for each specimen had to be entered to the universal testing machine software database, so the tensile stress could be calculated. The key issue of the conducted work was the comparative analysis of what was completed for all sample models without extensometer.

### 2.5. Static Tensile Strength Test

The static tensile strength tests were carried out at a displacement rate of 1 mm/min using an Inspekt mini 3kN universal testing machine (Hegewald & Peschke MPT GmbH, Nossen, Germany). The specimens were placed in a clamping handle and then preloaded in accordance with the measurement procedure; after stabilizing the pressure, the test was started.

### 2.6. SEM Microscopy Analysis

A JEOL JSM-7100F scanning electron microscope (SEM, JEOL, Tokyo, Japan) was employed to observe the cross-sectional area of the specimens after fracture. Magnifications from ×20 to ×2000 were used for the analysis. The photographs were directly taken for the tested surface without the need to carry out the sputtering process, which increases the quality of the measurement results. The photos show an example of measuring the thickness of the samples and the diameters of the grains of unsintered powder.

### 2.7. Specimens Nomenclature

Prior to the static tensile strength tests, parallel lines were drawn at 5 mm intervals along the gauge length, so the plastic deformation after failure could be easier to assess. The tests were conducted using five specimens per thickness.

The specimen identification number contained the build direction, specimen thickness, and the number in the measurement series. For example, the symbol X-0.35-1 stands for specimen 1 in the series of specimens with a thickness of 0.35 mm built in the X direction.

## 3. Results and Discussion

### 3.1. Tensile Test Results

**Figure 5:**
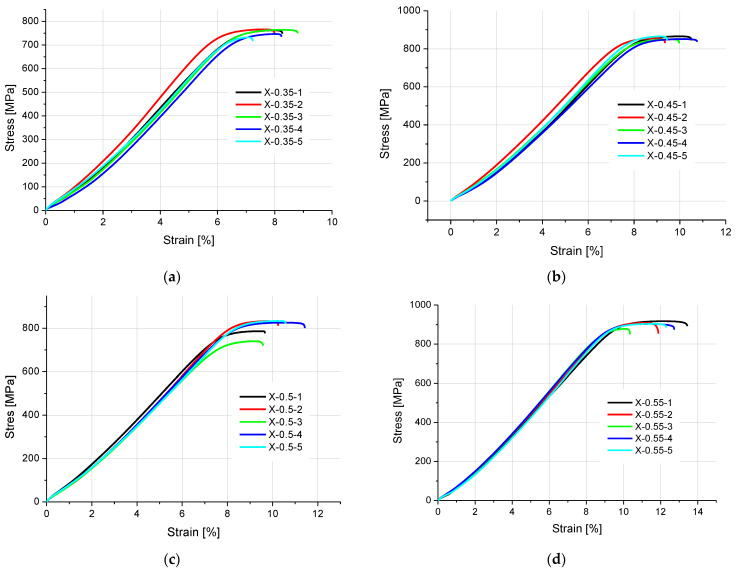
Stress–strain diagrams for the specimens with a thickness of (**a**) 0.35 mm, (**b**) 0.45 mm, (**c**) 0.5 mm, and (**d**) 0.55 mm, built in the X direction.

**Figure 6:**
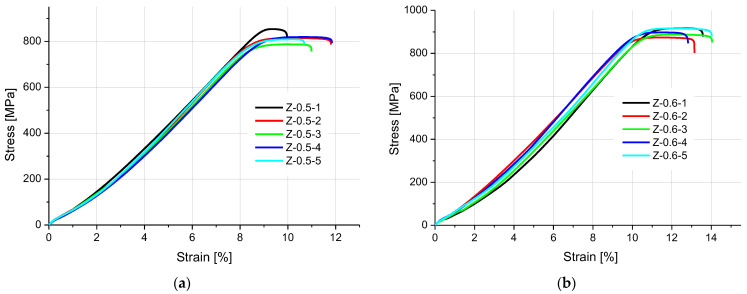
Stress–strain curves plotted for the specimens with a thickness of (**a**) 0.5 mm and (**b**) 0.6 mm, built in the Z direction.

The stress–strain curves are compared in Figure 5 and Figure 6.

**Figure 7:**
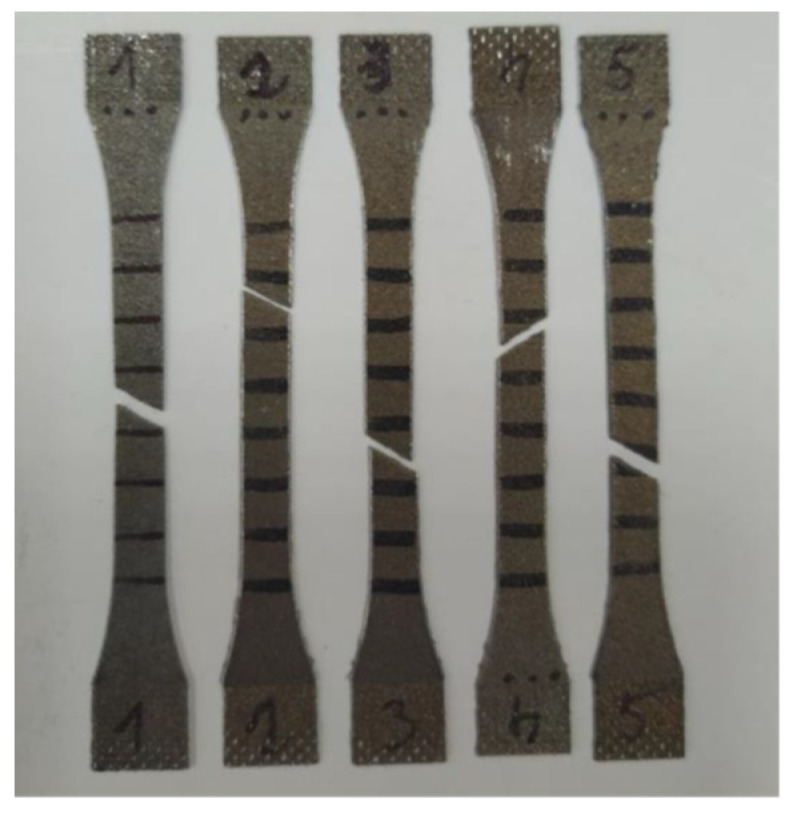
Example series of specimens after the static tensile strength tests.

Figure 7 shows a series of specimens after failure. As can be seen, they all broke at an angle of about 45°, i.e., in the direction of the maximum tangential stress. Rupture phenomena of this type were described, for instance, in refs. [7,27,28,29].

The ultimate tensile strength, R_m_, and the percentage of elongation after fracture, ε, determined in the experiments are provided in Table 3. The percentage of elongation after fracture, especially for metal alloys, characterizes the ability of a material to undergo permanent deformation.

**Figure 8:**
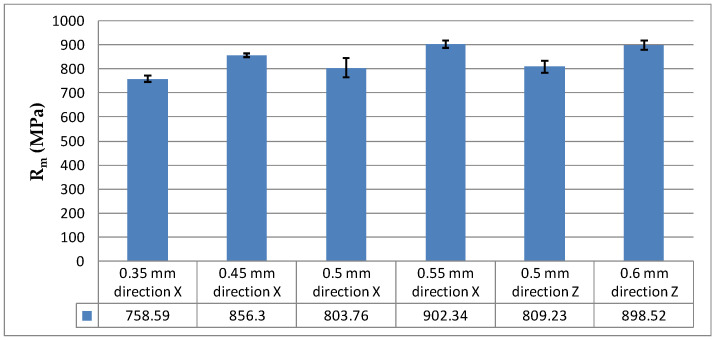
Tensile strength, R_m_, depending on the specimen thickness and build direction.

**Figure 9:**
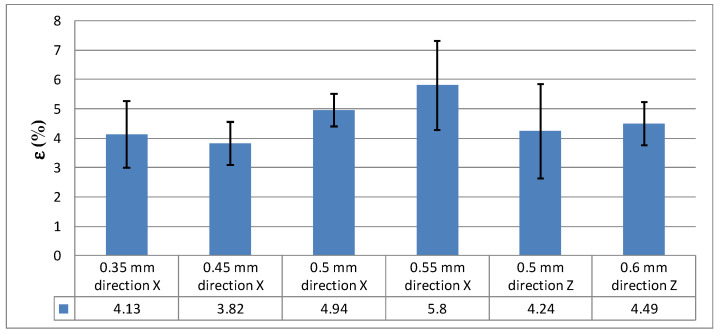
Percentage of elongation after fracture, ε, depending on the specimen thickness and build direction.

Bar charts were used to better illustrate the test results, i.e., the average values of the studied properties (Figure 8 and Figure 9).

From the experiments, it is clear that the mechanical properties of the thin-walled specimens were worse than those of the solid specimens that are used in standardized tests. The thickness was reported to have a crucial effect. Since standardized tests are performed using specimens with a much higher thickness, the design of thin-walled elements requires some necessary corrections; for example, the safety factor is increased several times. The tensile strength and the other properties of specimens fabricated by DMLS are not largely affected by the build direction, i.e., the specimen orientation on the build platform. When parts are additively manufactured by other technologies, there is clear anisotropy in their mechanical properties, which is dependent on the build direction. The previous research by the authors included studies of the mechanical behavior of thin-walled specimens fabricated by SLS from polyamide-powder-based composites as well as PLA-based composites [2,3,4]. The results presented in this article indicate that DLMS technology does not generate significant build-direction-dependent anisotropy for thin-walled elements.

The tensile strength of the thicker specimens (0.6 mm and 0.55 mm) was slightly higher than that of the other specimens. The tensile strength was about 19% higher for a specimen thickness of 0.55 mm than for 0.35 mm. A similar relationship, i.e., an increase in the tensile strength with increasing specimen thickness, was discussed in [6].

Analyzing the results of the tensile strength of thin-walled models, it can be concluded that their lower properties may partly result from the technological parameters, which in the manufacturing process are assumed to be the same as those of solid models. It seems that the key in this area, in the case of the production of thin-walled models, was the correction for the parameters of the DownShin module, which was characterized by the delivery of an over two-times-lower energy density compared to the Infill module; this value was, respectively, 25 J/mm^3^ and 54.91 J/mm^3^. In addition, the number of UpShin and DownShin layers was two and five, respectively, which in the case of thin-walled models, for example, flat prints (parallel with the plane of the table), may affect their strength due to an over two-times-lower value of energy density (DownSkin).

### 3.2. Microscopy

**Figure 10:**
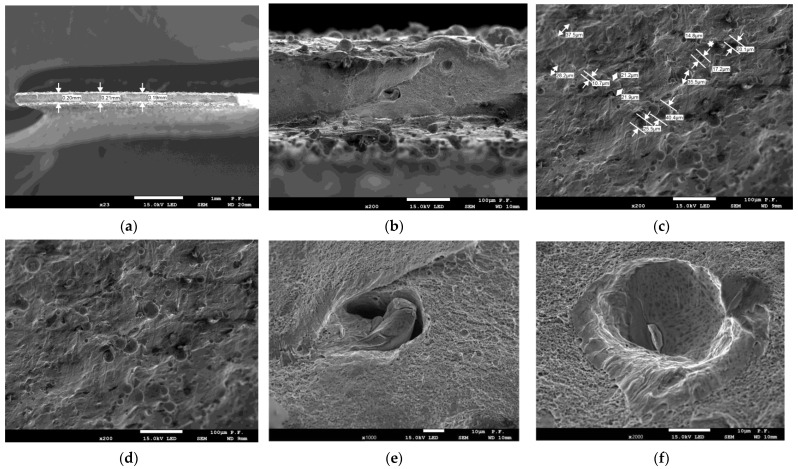
SEM images of a thin-walled specimen after fracture: (**a**) cross-section—magnification 23×, thickness measured at three points; (**b**) cross-section—magnification 200×; (**c**,**d**) side views—magnification 200×, unmolten powder grains at the surface differing in diameter; (**e**) local void—magnification 1000×; (**f**) crater with an inclusion—magnification 2000×.

**Figure 11:**
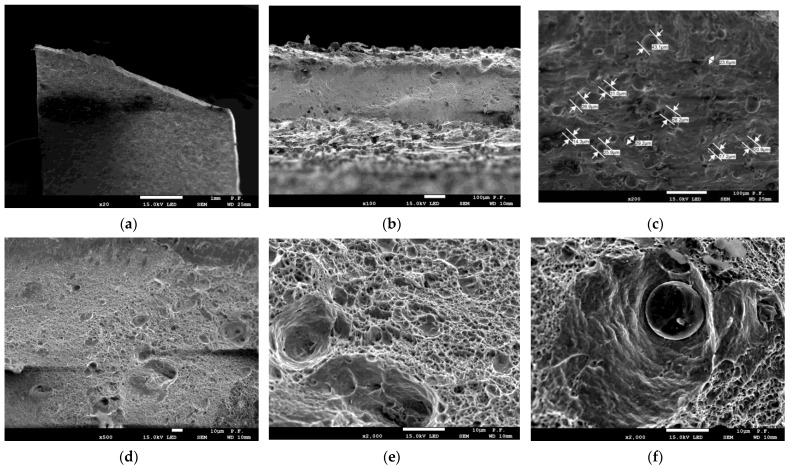
Vertically built maraging steel specimen after fracture: (**a**) side view—magnification 20×; (**b**) cross-section—magnification 200×; (**c**) side view—magnification 200×, measured diameters of unmolten powder grains; (**d**) side view—magnification 500×; (**e**) side view—magnification 2000×; (**f**) unsintered grain—magnification 2000×.

The microscopy results using SEM are presented in images and shown in Figure 10 and Figure 11.

From the measurement of the cross-sectional area of the specimens after fracture (Figure 10a), it was evident that classical necking (plastic deformation) had occurred. In the case of thin-walled specimens, this could not be seen with the naked eye. The difference in the cross-sectional area between the as-printed and deformed specimens was 0.15 mm. Figure 10b depicts spherical unmolten powder grains. Slight melting took place only at the grain boundaries; this was why there were unsintered grains at the specimen surface, which were not removed while the specimens were cleaned. As shown in Figure 10c, some of the diameters of unmolten powder grains were measured; they were in the range of 14.3–43.1 µm. The local flaws in the macrostructure of the molten material visible in Figure 10e,f were likely to affect the mechanical properties of parts. Their influence was particularly high under changeable loading conditions, as described in [7,18,30]. The results of the microscopic analysis depicted in Figure 10b and Figure 11b, i.e., the SEM images of the unmolten powder grains found at the specimen surface, were similar to the those discussed in [30] (in both cases, magnification 200×).

The unmolten grains, which are visible in Figure 11c, ranged between 14.3 µm and 43.1 µm in diameter. Those with a diameter ranging from 20.6 µm to 26.9 µm were the most numerous. Figure 11f shows an unsintered powder grain as an inclusion in a crater formed during fusion; it must have entered the pool from the outside, as it was not affected by the energy of the laser beam.

## 4. Conclusions

The major findings of this study, which was concerned with the thin-walled specimens printed by DMLS from EOS MaragingSteel MS1 powder, are as follows:

- there was no significant anisotropy in the mechanical properties of the elements printed in different directions;
- the tensile strength increased with increasing thickness, and it was approximately 19% higher for the thickest elements than for the thinnest ones;
- due to a high standard deviation (SD) and a wide scatter of the results for the percentage of elongation after fracture, ε, it was difficult to accurately determine the relationship between the two parameters;
- elements printed in the X direction were characterized by the highest repeatability, as indicated by the close to zero values of SD in Table 2;
- the analysis of the technological process for standard technological parameters showed that for thin-walled models it was necessary to introduce a correction in the number of *DownSkin* layers, which resulted in an increase in the energy density.

## Figures and Tables

**Table 1:** Mechanical properties of EOS MaragingSteel MS1 [26].

Mechanical Property ^1^	As Built ^2^	
	Horizontal	Vertical
Ultimate tensile strength, R_m_	1200 MPa	1200 MPa
Yield strength, Rp0.2	1020 MPa	1050 MPa
Elongation at break, A	13%	11%
	Heat Treated ^3^	
	Horizontal	Vertical
Ultimate tensile strength, R_m_	2060 MPa	2080 MPa
Yield strength, Rp0.2	1990 MPa	2010 MPa
Elongation at break, A	4%	3%

^1^ Tensile testing according to ISO 6892-1 B10, proportional test pieces, diameter of the neck area 5 mm, original gauge length 25 mm. ^2^ The numbers are average values determined from samples with horizontal and vertical orientations. ^3^ Heat treatment procedure: aging temperature 490 °C for 6 h, air cooling.

**Table 2:** Dimensions of the MaragingSteel MS1 specimens before the tensile tests.

**Specimens** **X-0.35**	$\bar{a}$ **(mm)**	$\bar{b}$ **(mm)**	**Specimens** **X-0.45**	$\bar{a}$ **(mm)**	$\bar{b}$ **(mm)**	**Specimens** **X-0.5**	$\bar{a}$ **(mm)**	$\bar{b}$ **(mm)**
1	0.36	5.17	1	0.45	5.15	1	0.50	5.46
2	0.36	5.00	2	0.45	5.17	2	0.49	5.31
3	0.36	5.17	3	0.45	5.22	3	0.52	5.37
4	0.35	5.11	4	0.45	5.31	4	0.50	5.19
5	0.36	5.06	5	0.45	5.16	5	0.50	5.18
$\bar{x}$	0.36	5.10	$\bar{x}$	0.45	5.20	$\bar{x}$	0.50	5.30
*SD*	0.00	0.07	*SD*	0.00	0.06	*SD*	0.01	0.11
**Specimens** **X-0.55**	$\bar{a}$ **(mm)**	$\bar{b}$ **(mm)**	**Specimens** **Z-0.5**	$\bar{a}$ **(mm)**	$\bar{b}$ **(mm)**	**Specimens** **Z-0.6**	$\bar{a}$ **(mm)**	$\bar{b}$ **(mm)**
1	0.55	5.17	1	0.51	5.17	1	0.57	5.19
2	0.55	5.15	2	0.49	5.24	2	0.58	5.34
3	0.55	5.16	3	0.51	5.22	3	0.57	5.30
4	0.55	5.14	4	0.49	5.36	4	0.57	5.29
5	0.55	5.19	5	0.49	5.32	5	0.56	5.37
$\bar{x}$	0.55	5.16	$\bar{x}$	0.50	5.26	$\bar{x}$	0.57	5.30
*SD*	0.00	0.02	*SD*	0.01	0.07	*SD*	0.01	0.06

**Table 3:** Mechanical properties of thin-walled specimens (MaragingSteel MS1).

**No.** **X-0.35**	***R_m_*** **(MPa)**	***ε*** **(*%*)**	**No.** **X-0.45**	***R_m_*** **(MPa)**	***ε*** **(*%*)**	**No.** **X-0.5**	***R_m_*** **(MPa)**	***ε*** **(*%*)**
1	762.32	4.46	1	859.53	4.43	1	786.57	5.14
2	766.19	2.54	2	855.34	2.74	2	832.77	4.29
3	764.28	4.23	3	850.16	3.80	3	740.21	5.43
4	767.92	5.66	4	851.24	4.54	4	825.85	4.29
5	732.22	3.74	5	865.21	3.57	5	833.39	5.57
$\bar{x}$	758.59	4.13	$\bar{x}$	856.30	3.82	$\bar{x}$	803.76	4.94
*SD*	14.89	1.13	*SD*	6.21	0.73	*SD*	40.44	0.55
**No.** **X-0.55**	***R_m_*** **(MPa)**	***ε*** **(*%*)**	**No.** **Z-0.5**	***R_m_*** **(MPa)**	***ε*** **(*%*)**	**No.** **Z-0.6**	***R_m_*** **(MPa)**	***ε*** **(*%*)**
1	917.29	7.34	1	853.68	1.46	1	917.25	3.43
2	908.98	6.83	2	814.87	4.86	2	873.56	5.43
3	878.06	3.51	3	787.25	4.34	3	887.78	4.77
4	903.36	6.14	4	819.01	5.49	4	897.61	4.26
5	904.03	5.20	5	809.23	5.03	5	916.42	4.57
$\bar{x}$	902.34	5.80	$\bar{x}$	816.80	4.24	$\bar{x}$	898.52	4.49
*SD*	14.67	1.51	*SD*	23.98	1.61	*SD*	18.78	0.73

## Data Availability

Not applicable.
